# Pore Structure Changes Occur During CO_2_ Injection into Carbonate Reservoirs

**DOI:** 10.1038/s41598-020-60247-4

**Published:** 2020-02-27

**Authors:** Mojtaba Seyyedi, Hisham Khaled Ben Mahmud, Michael Verrall, Ausama Giwelli, Lionel Esteban, Mohsen Ghasemiziarani, Ben Clennell

**Affiliations:** 1 Australian Resources Research Centre, CSIRO, Kensington, Australia; 2 Curtin University Malaysia, CDT 250, 98009 Miri Sarawak, Malaysia; 3 Curtin University, Bentley, Australia

**Keywords:** Chemical engineering, Energy, Carbon capture and storage

## Abstract

Observations and modeling studies have shown that during CO_2_ injection into underground carbonate reservoirs, the dissolution of CO_2_ into formation water forms acidic brine, leading to fluid-rock interactions that can significantly impact the hydraulic properties of the host formation. However, the impacts of these interactions on the pore structure and macroscopic flow properties of host rock are poorly characterized both for the near-wellbore region and deeper into the reservoir. Little attention has been given to the influence of pressure drop from the near-wellbore region to reservoir body on disturbing the ionic equilibrium in the CO_2_-saturated brine and consequent mineral precipitation. In this paper, we present the results of a novel experimental procedure designed to address these issues in carbonate reservoirs. We injected CO_2_-saturated brine into a composite core made of two matching grainstone carbonate core plugs with a tight disk placed between them to create a pressure profile of around 250 psi resembling that prevailing in reservoirs during CO_2_ injection. We investigated the impacts of fluid-rock interactions at pore and continuum scale using medical X-ray CT, nuclear magnetic resonance, and scanning electron microscopy. We found that strong calcite dissolution occurs near to the injection point, which leads to an increase in primary intergranular porosity and permeability of the near injection region, and ultimately to wormhole  formation. The strong heterogeneous dissolution of calcite grains leads to the formation of intra-granular micro-pores. At later stages of the dissolution, the internal regions of ooids become accessible to the carbonated brine, leading to the formation of moldic porosity. At distances far from the injection point, we observed minimal or no change in pore structure, pore roughness, pore populations, and rock hydraulic properties. The pressure drop of 250 psi slightly disturbed the chemical equilibrium of the system, which led to minor precipitation of sub-micron sized calcite crystals but due to the large pore throats of the rock, these deposits had no measurable impact on rock permeability. The trial illustrates that the new procedure is valuable for investigating fluid-rock interactions by reproducing the geochemical consequences of relatively steep pore pressure gradients during CO_2_ injection.

## Introduction

Capturing CO_2_ from large industrial sources and storing it in geological formations, such as saline aquifers, and depleted oil and gas fields, has become widely accepted as a viable solution for reducing high CO_2_ levels in the atmosphere^1–3^. Among underground formations, carbonate reservoirs are attractive CO_2_ sequestration options, as the majority of the world’s oil reserves (60%) are held in these types of rocks (especially in the Middle East), making them a primary storage target when combined with enhanced oil recovery (EOR) operations^4^. Furthermore, since these reservoirs could hold the hydrocarbons for millions of years, it can be assumed that they can hold the CO_2_ for an indefinite period through a combination of physical and chemical trapping mechanisms. However, injected CO_2_ mixes with the formation water to produce an acidic brine (i.e., CO_2_-saturated brine) that reacts with carbonate minerals and can affect formation integrity, injectivity, and consequently the practicality of safe CO_2_ storage^5–7^. Moreover, storage capacity and dynamics of the injected CO_2_ plume can be affected by changes in rock porosity and permeability^8,9^. Therefore, investigating CO_2_-water-carbonate rock interactions prior to CO_2_ injection into carbonate reservoirs is important.

Numerous studies have investigated the interactions between CO_2_-saturated brine and carbonate rocks in laboratory core flooding tests^10–20^. However, the majority of these studies only report the alterations in rock permeability and porosity in a general sense. Less attention has been given to the impact of geochemical reactions on rock pore structure, pore size, pore size distributions, pore body roughness, and their consequent impact on porosity type, fluid trapping, and CO_2_ storage capacity.

During CO_2_ injection into saline aquifers or depleted oil fields, a CO_2_-saturated brine (carbonated brine) front will be formed ahead of the CO_2_ front. Since during the injection, the injection pressure or near wellbore pressure can be several 100 psi higher than reservoir pressure, the carbonated brine has a saturation pressure higher than the reservoir pressure. As carbonated brine flows into reservoir, it reacts with carbonate minerals and becomes enriched with Ca^2+^ and HCO_3_^− ^ions. At regions far from the injection well or when injection is stopped^21–23^, the pressure of the carbonated brine front decreases. This pressure drop alters the chemical equilibrium between the dissolved species leading to calcite precipitation. Such precipitation can impact the hydraulic properties of the rock and may even lead to the sealing of flow paths; therefore, its study is of importance. However, the majority of published studies report core flooding tests with a pressure gradient negligible compared to those commonly encountered in a field situation. Therefore, the potential impacts of mineral precipitation and consequent changes on rock porosity, permeability, pore structure, pore size, pore size distributions, and pore body roughness have likely been underestimated hitherto.

We address these gaps in knowledge using a novel core flooding approach that examines effects from pore to continuum scales. A composite core sample was made of two outcrop carbonate core plugs with a high entry pressure, low permeability ceramic disk placed between them. The tight disk provided a high-pressure gradient in the system resembling that in the near-wellbore region during CO_2_ injection into a reservoir. The composite core was flushed by carbonated brine for a specified number of pore volumes and its hydraulic properties and pore structure were investigated prior to and following the carbonated brine injection step where the fluid-rock interactions occurred.

## Materials

### Fluids

A high salinity brine composed of 73 g/l NaCl in water was used in this study. The total salinity of this brine resembles the high salinity of formation brines in carbonate reservoirs in the North Sea^24^ and the Middle East^25^. To make the carbonated brine, CO_2_ with a purity of 99.99 mol% was mixed with the brine at test conditions (2500 psi and 50 °C) using a rocking cell. According to the Duan *et al*.^26^. Equation of State, the CO_2_ solubility at these conditions in the brine is around 22 scc CO_2_/scc brine.

### Cores

Two Savonnieres limestone cores were obtained from a quarry in the North-East of France (Lorraine region). Savonnieres limestone is a widely used standard carbonate rock used in many published studies of petrophysical and flow properties^27–31^. The XRD results of the rock show that the cores are almost entirely composed of calcite (>99%). The cores properties are shown in Table 1. Initial cores porosities were measured using helium while absolute permeabilities were measured using the brine at the experimental conditions (2500 psi and 50 °C).

**Table 1:** Cores properties and dimensions.

Core	Length (cm)	Diameter (cm)	Gas porosity (%)	Brine permeability (mD)
A	8.1	3.8	24.9	39.4
B	8	3.8	28.6	43.2

## Experimental Setup

### Core flood rig

**Figure 1:**
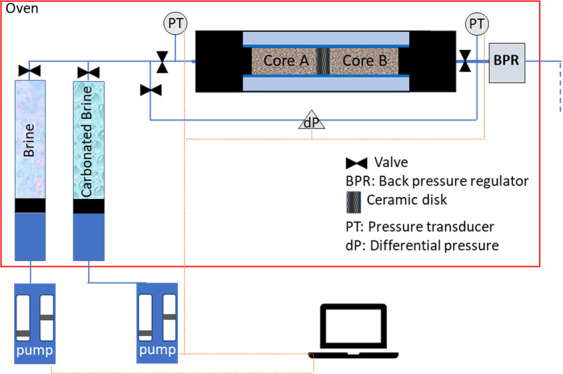
Simple schematic of high-pressure and -temperature core flooding rig.

A high-pressure and high-temperature core flooding system was used the schematic of which is shown in Fig. 1. The main components of the rig are high-pressure pumps, fluid cells, core holder, back pressure regulator, and an oven.

### Medical X-ray CT

A medical X-ray CT scanner (Siemens Somatom Definition AS) with an X-ray beam of 140 kV and 1000 mAs was used for scanning the cores with the aim of studying any changes in CT attenuation number of the cores due to fluid-rock interactions (either dissolution or precipitation). The obtained voxel resolution was ~120 µm × 120 µm in the plane of the slices x 400 µm slice thickness. Avizo software (Thermofisher) was used on the 512 ×512 pixels CT images to extract the mean X-ray CT attenuation along the core axis which was then transformed into equivalent bulk density. To measure the mean CT numbers along the core length, a circular region of interest with an area of 8.15 cm^2^ to cover the core plug surface but avoiding edge pixels was selected for each transversal CT image.

### Nuclear magnetic resonance

The general pore structures of the core plugs were studied using low-frequency Nuclear Magnetic Resonance (NMR) on brine-saturated core plugs. NMR relaxation signal intensity of a fluid-saturated porous media can provide insights into the pore surface area, pore volume, and pore size populations and distribution. NMR T_2_ can be derived from:

$$\frac{1}{{T}_{2}}=\frac{1}{{T}_{2B}}+\rho \,\frac{S}{V}$$

Where $\rho$ refers to the surface relaxivity parameter, T_2B_ refers to the relaxation time of bulk water, S is the pore surface area, and V is pore volume. In many cases, the relaxation time of water in porous media is much smaller than that of bulk water as such T_2_ can be derived from:

$$\frac{1}{{T}_{2}}\approx \rho \,\frac{S}{V}$$

Pore surface area is a direct function of pore roughness. Therefore, according to the above equation, an increase in pore surface area or pore roughness leads to a decrease in NMR T_2_ relaxation time while an increase in pore volume leads to an increase in NMR T_2_ relaxation time. In this study, a 2.37 MHz Geospec2 NMR spectrometer system from Oxford-GIT (Ltd.) was used with a 53 mm probe Q-sense (max. gradient strength of 50 G/cm) to obtain transverse relaxation (T_2_) decay from CPMG spin-echo sequence as well as saturation profile along the core length. The echo-spacing is about 140 µs (Tau = 57 µs) with a receiving delay of 7,500 ms.

## Experimental Procedure

### Baseline- Brine flooding

Each core was first fully saturated with the brine at experimental conditions (2500 psi and 50 °C) and its brine absolute permeability was measured. The brine-saturated cores were then scanned to obtain the mean CT attenuation number along the length of the cores. The fully brine-saturated cores were taken out of the core holder to immediately initiate the NMR T_2_ and saturation profile measurements with the aim of characterizing the original pore size distribution. Next, brine-saturated cores were loaded into the core holder with a tight ceramic disk placed between them to create a high-pressure gradient across the composite core during flooding steps. At the injection rate of 10 cc/h, the tight disk provides a pressure gradient of around 300 psi along the length of composite core and a local pressure drop of around 250 psi after the disk. Core plugs A and B made the inlet and outlet parts of the composite core, respectively. With this configuration, Core A represents the near injection well regions where the pressure is higher than reservoir pressure and Core B represents regions far from the injection well where the pressure is equal to reservoir pressure. The back pressure was set at 2200 psi and the composite core was flooded with the brine to measure its absolute permeability.

### Carbonated brine injection

The composite core was then flooded by the carbonated brine at the rate of 10 cc/h for 10 pore volumes (around 50 h). During this period, the pressure gradient across the composite core was recorded while the geochemical reactions between CO_2_-saturated brine-minerals occur. Given the low flow rate of carbonated brine, it has a considerable contact time with Core A to get enriched in Ca^2+^ and HCO_3_^−^ ions prior to flowing through the tight disk and Core B. For the used composite core under the test conditions, the estimated values of Péclet and diffusive Damköhler numbers are 11.4 and 4.6e-2 using the equations presented by Gray *et al*.^32^ and the reaction rate of 0.001 mol/m^2^.sec taken from Plummer *et al*.^33^. The high Péclet number indicates material transport dominated by advection, while the low value of Damköhler number indicates that the calcite dissolution rate during fluid-rock interaction is limited by reaction kinetics and is not limited by diffusive mass transfer.

### Repeat brine flooding

After the carbonated brine injection step, without changing any experimental conditions, brine was injected into the composite core at a lower rate of 5 cc/h for around 10 pore volumes. A lower rate was chosen to be sure that possible precipitates in the cores will not be displaced by the hydrodynamic drag force. During this step, the pressure gradient across the composite core was measured to calculate the mean absolute permeability of the system after the CO_2_-saturated brine-rock interactions. The brine-saturated cores were then scanned to obtain the mean CT attenuation number along the length of cores. The fully brine-saturated cores were taken out of the core holder to immediately initiate the NMR T_2_ and saturation profile measurements. The cores’ permeabilities were then separately measured. Finally, scanning electron microscope (SEM) images of the cores A and B inlet faces were taken before and after the carbonated brine injection step to gain further insights into pore-scale changes.

## Results and Discussion

### Injectivity

**Figure 2:**
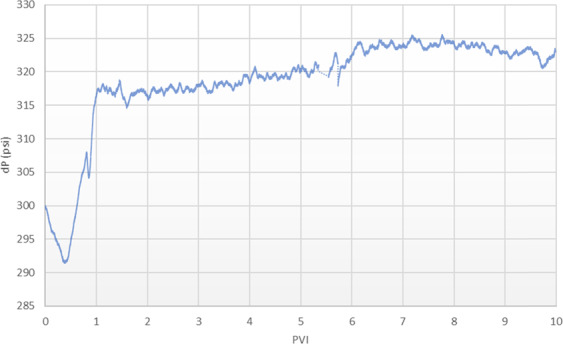
Differential pressure (dP) across the composite core during the carbonated brine injection step.

The pressure gradient across the composite core during the carbonated brine injection step is shown in Fig. 2. As soon as the carbonated brine front meets the inlet face of the composite core (i.e., Core A), the pressure gradient across the composite core decreased slightly. This may be due to mineral dissolution within Core A that increases its pore connectivity and thus permeability. As the carbonated brine front meets the low permeability disk, the pressure gradient started to sharply increase up to almost one pore volume of injection (PVI), and after that, the increase continued more slowly. One possible  reason for such a sharp increase in the pressure gradient across the composite core is the exsolution of CO_2_ from the carbonated brine front due to the local pressure drop of 250 psi after passing the tight disk. This caused the system to shift from single-phase flow to 2-phase flow. The presence of CO_2_ as a free phase creates resistance for water to flow and therefore increases the pressure gradient.

The comparison of composite core’s absolute permeability to the brine before and after the carbonated brine injection step showed around 4% reduction in its absolute permeability. To understand this reduction, the permeability of each core to the brine after the carbonated brine injection step was separately measured and the results were compared with the original values. According to results, the permeability of Core A was increased by 175% while the permeability of Core B remained almost unchanged. These results show that the reduction in composite core permeability to the brine was due to the reduction in the permeability of the disk as a result of calcite precipitation at the outlet face of the disk. This calcite precipitation originates from the sudden local pressure drop in the outlet face of the tight disk and is discussed in further detail in the next sections. Furthermore, due to the carbonated brine rock interactions, the porosity of Core A was increased by 6% while the porosity of the Core B remained almost unchanged.

### Pore structure variations

To identify changes in the pore structure of each core plug due to carbonated brine-rock interactions, CT scan images, NMR T_2_ profiles, NMR saturation profiles, and SEM images of each core were taken before and after carbonated brine injection step and results were compared.

#### Core A

**Figure 3:**
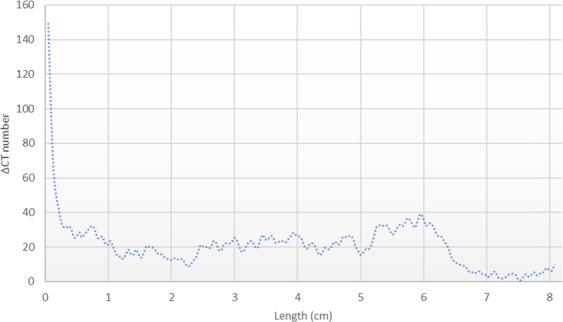
X-ray CT number gradient along the Core A from the inlet side (left) to outlet side (right) shows a strong mineral dissolution (ΔCT > 0), especially at the inlet side.

Figure 3 shows the gradient of X-ray CT attenuation number across the length of Core A which was obtained by:

$$\Delta {\rm{CT}}\,{\rm{number}}={\rm{CT}}\,{\rm{original}}-{\rm{CT}}\,{\rm{after}}\,{\rm{carbonated}}\,{\rm{brine}}\,{\rm{injection}}\,{\rm{step}}$$

Therefore, ΔCT > 0 refers to mineral dissolution, ΔCT < 0 refers to mineral precipitation, and the higher absolute value of ΔCT shows the stronger mineral dissolution or precipitation. Figure 3 shows the strong mineral dissolution that occurred in Core A due to the carbonated brine injection step. The dissolution is stronger near the Core A inlet face region than the outlet. This is because plenty of fresh acidic brine (or carbonated brine) came in contact with the inlet face. This is resembling the near-wellbore region where significant pore volumes of carbonated brine meet the formation rock. Therefore, it is expected that severe mineral dissolution takes place in this region which can impact the rock mechanical properties and wellbore integrity.

**Figure 4:**
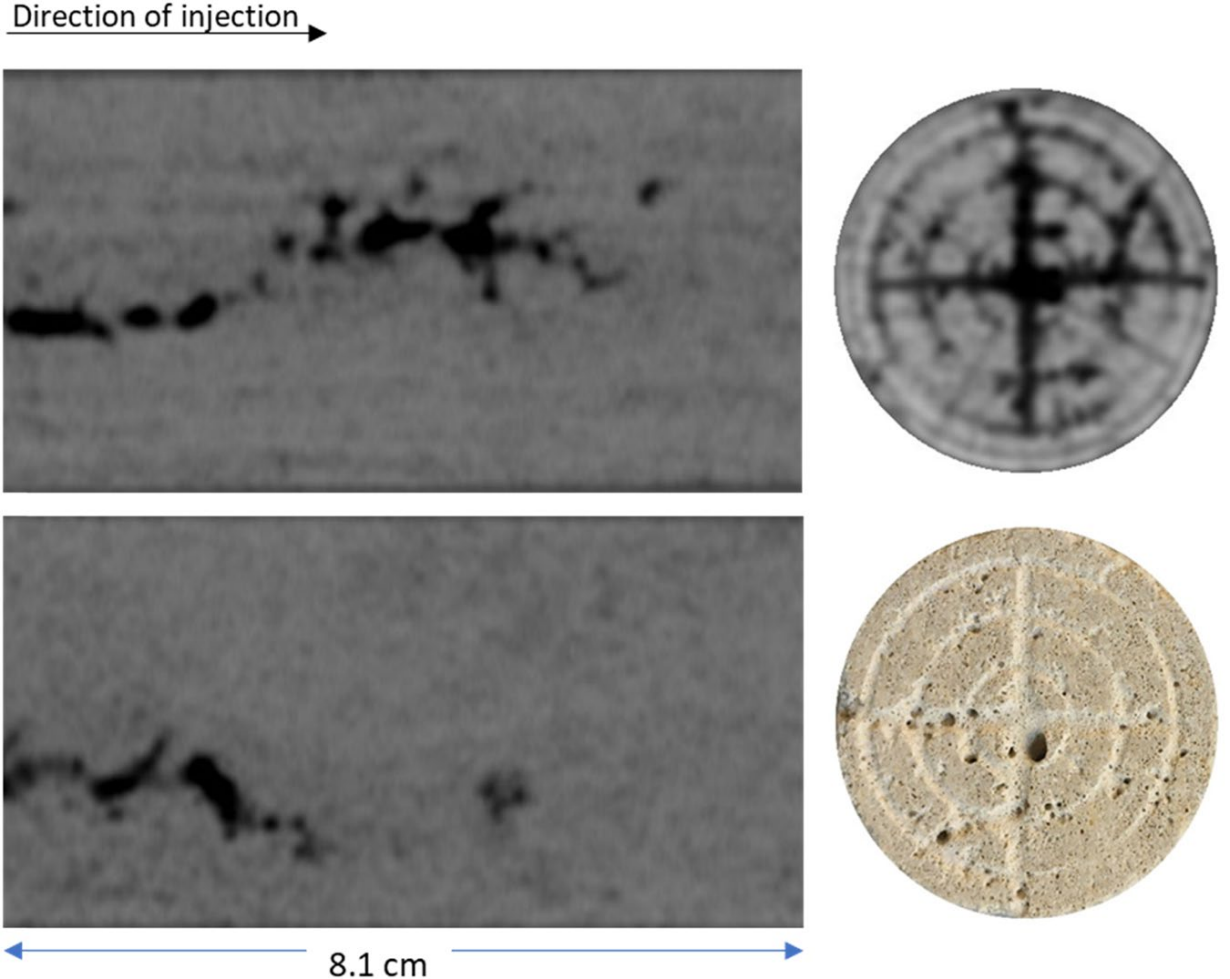
X-ray CT images of the Core A after carbonated brine injection. Two orthogonal longitudinal views from the inlet (left) to the outlet (right) and a transversal view at the near inlet face of the core.

Figure 4 illustrates the X-ray CT images taken from the inlet side and two orthogonal cross-sections along the length of Core A after the carbonated brine injection step. The dark areas in this figure represent regions in the core where the calcite dissolution occurred in the presence of carbonated brine. This figure also shows the image of the inlet face of the Core A fter the carbonated brine injection step. The dissolution led to the formation of wormholes.

**Figure 5:**
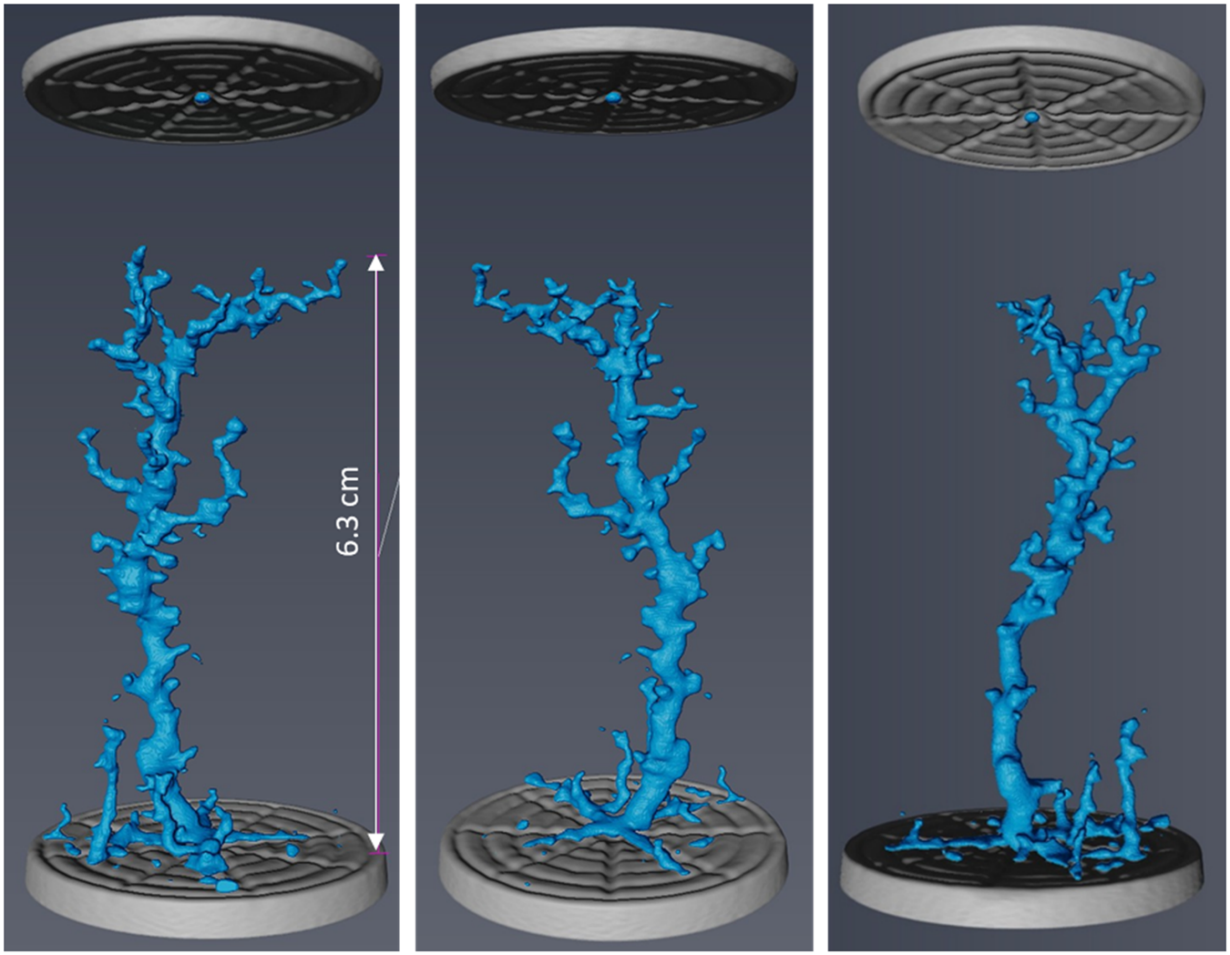
Dissolution induced wormholes in Core A viewed from thee different angles. The grey discs at the top and bottom of the images are metal platens used for injecting fluids. The injection direction was from the bottom to top of the images.

Avizo software (Thermofisher) was used to conduct 3D volume rendering on the CT images. Prior to image analysis, the image quality was improved using a Non-Local Means filter. Image segmentation was performed using a single threshold and watershed method on the resolved wormholes and solution pores. The results of segmentation are shown from three different angles in Fig. 5. The maximum penetration depth of wormholes is around 6.3 cm. The presence of dissolution-induced wormholes is the main reason behind the 175% increase in the absolute permeability of Core A after the carbonated brine injection step. Such a big wormhole acts as a conduit for fluids and significantly improves the fluid’s conductivity in the core.

**Figure 6:**
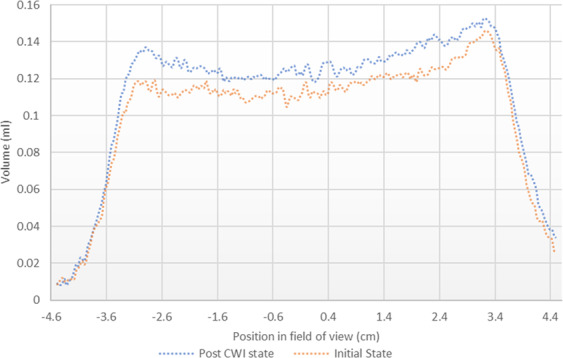
NMR T_2_ saturation profiles along the length of Core A before (orange curve) and after (blue curve) carbonated brine injection (CWI) step from left (inlet) to right (outlet). The post-CWI state profile shows an increase in pore volume due to calcite dissolution, especially at the inlet side.

**Figure 7:**
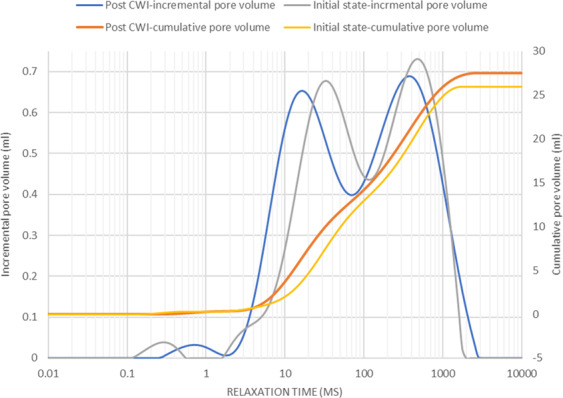
T_2_ relaxation time profiles of Core A before and after carbonated brine injection (CWI) step indicates the changes in pore size populations.

To study the impacts of calcite dissolution on the porosity, the NMR saturation profile along the length of Core A was measured before and after the carbonated brine injection step and the comparison is shown in Fig. 6. Consistent with X-Ray CT data (Figs. 3–5), the comparison of NMR saturation profiles shows that carbonated brine injection induced strong calcite dissolution which in turn increased the total pore volume or porosity of Core A. NMR T_2_ profiles of the Core A were also measured before and after the carbonated brine injection step and their comparison is shown in Fig. 7. NMR T_2_ of Core A at the initial state shows two main pore populations at around relaxation times of 20 and 500 ms. There is a small third pore population at around 0.3 ms that represents micro-pores. Calcite dissolution caused around 17% decrease in the population of micro-pores (T_2_ < 3 ms), 1.2% decrease in the population of large pores (100 < T_2_ < 1000 ms), while it led to 11% increase in the population of medium size pores (3 < T_2_ < 100 ms). Calcite dissolution also led to the enlargement of bigger pores, and consequently merged them together and formed wormholes (T_2_ > 1000 ms).

Furthermore, the calcite dissolution that occurred during the carbonated brine injection step shifted the main two pore populations toward smaller relaxation times while slightly shifting the population of micro-pores toward longer relaxation time. Calcite dissolution during carbonated brine injection caused an increase in the micro pore volume and increased the T_2_ relaxation time of this small pore population. However, for medium size pores (3 < T_2_ < 100 ms) calcite dissolution induced by carbonated brine injection led to an increase in pores roughness with little change in the volume of this pore size population: accordingly this peak shifts towards a shorter time. A similar but less pronounced trend was observed for large pores (100 < T_2_ < 1000 ms). We interpret this to mean that for macropores the ratio of increase in pore roughness to increase in pore volume during calcite dissolution was smaller than that of medium size pores. Large pores provide the preferential path for carbonated brine flow as such calcite dissolution and pore volume increase occurs stronger for them than medium sized pores. For the largest pores (T_2_ > 1000 ms) since the impact of calcite dissolution on increasing pore volume was much higher than any effects on pore roughness, the maximum relaxation times observed extend to somewhat larger values after the experiment.

**Figure 8:**
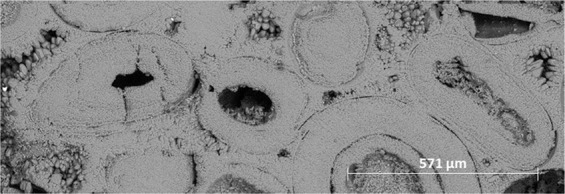
Oolitic grainstone with ooids cemented together via coarser calcite crystals.

**Figure 9:**
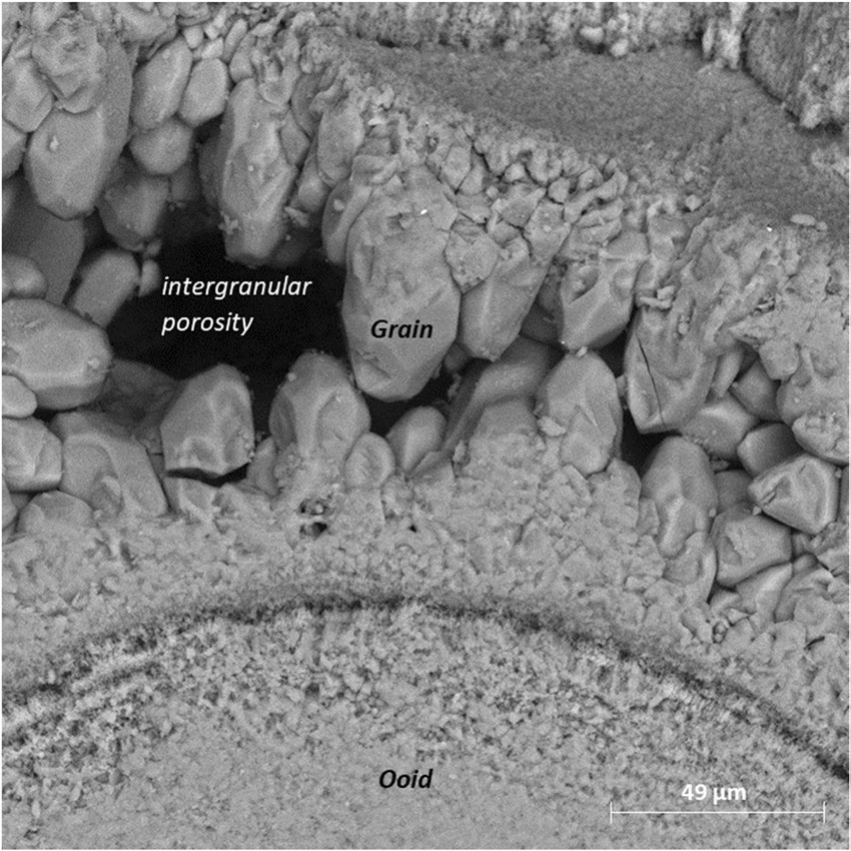
Intergranular porosity between rounded and angular calcite crystals surrounding the ooids.

To gain further insights into the pore structure alterations due to carbonated brin-rock interactions, SEM images at various scales were obtained from the inlet face of Core A. Figure 8 shows the pore structure of Core A in its initial state. The carbonate core is made of small (less than 450 µm in diameter) ooids which are cemented together by coarser calcite crystals forming an interconnected porous structure with primary intergranular porosity shown in Fig. 9. Each ooid is formed of a nucleus around which cortical layers of calcite are deposited to form a sub-spherical grain.

**Figure 10:**
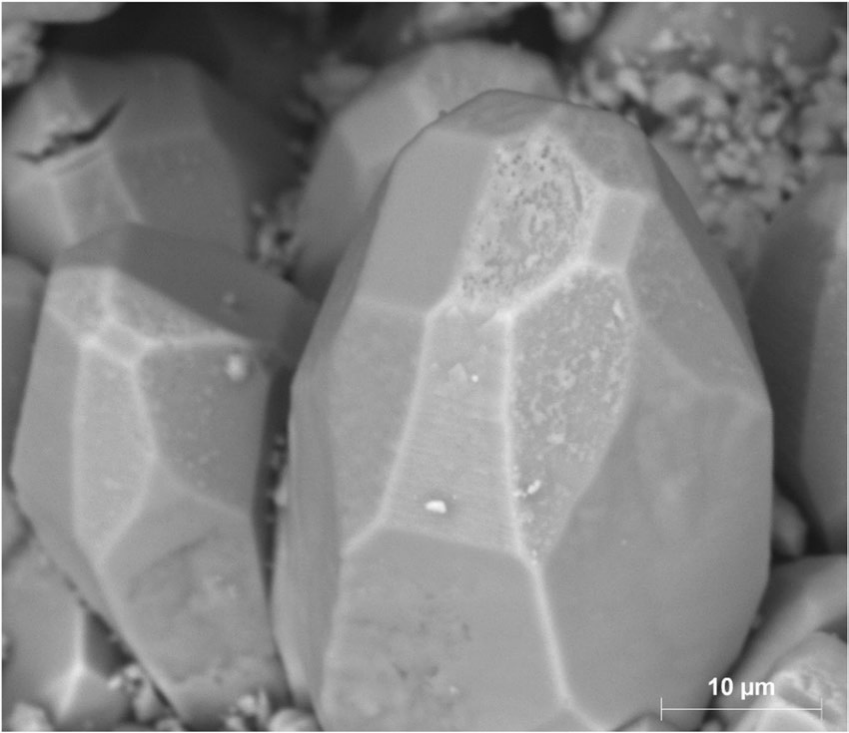
Rounded and angular calcite crystals.

**Figure 11:**
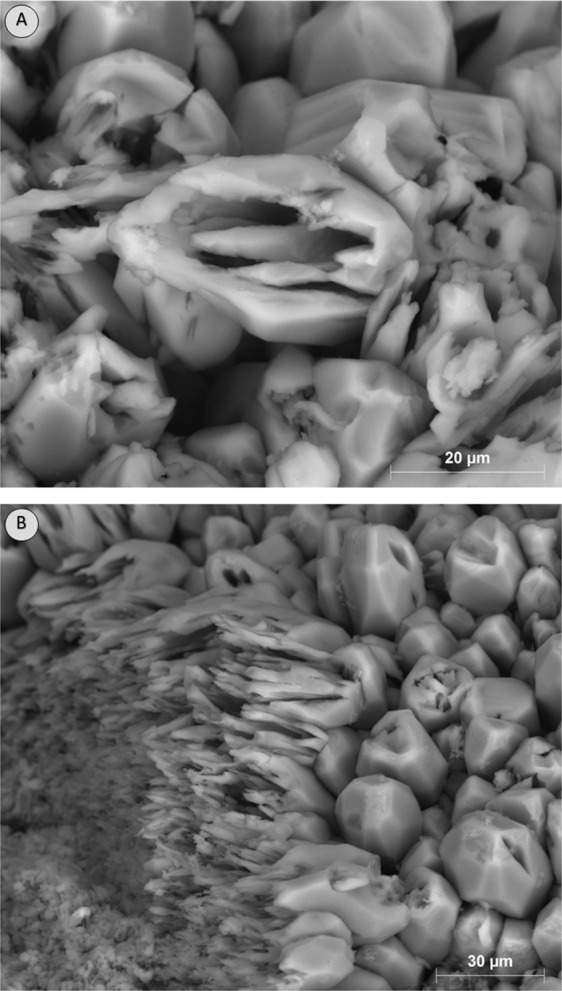
Heterogeneous dissolution of calcite grains (**A** and **B**) at the presence of carbonated brine increased the pore body roughness and formed intra-grain micro-porosity.

**Figure 12:**
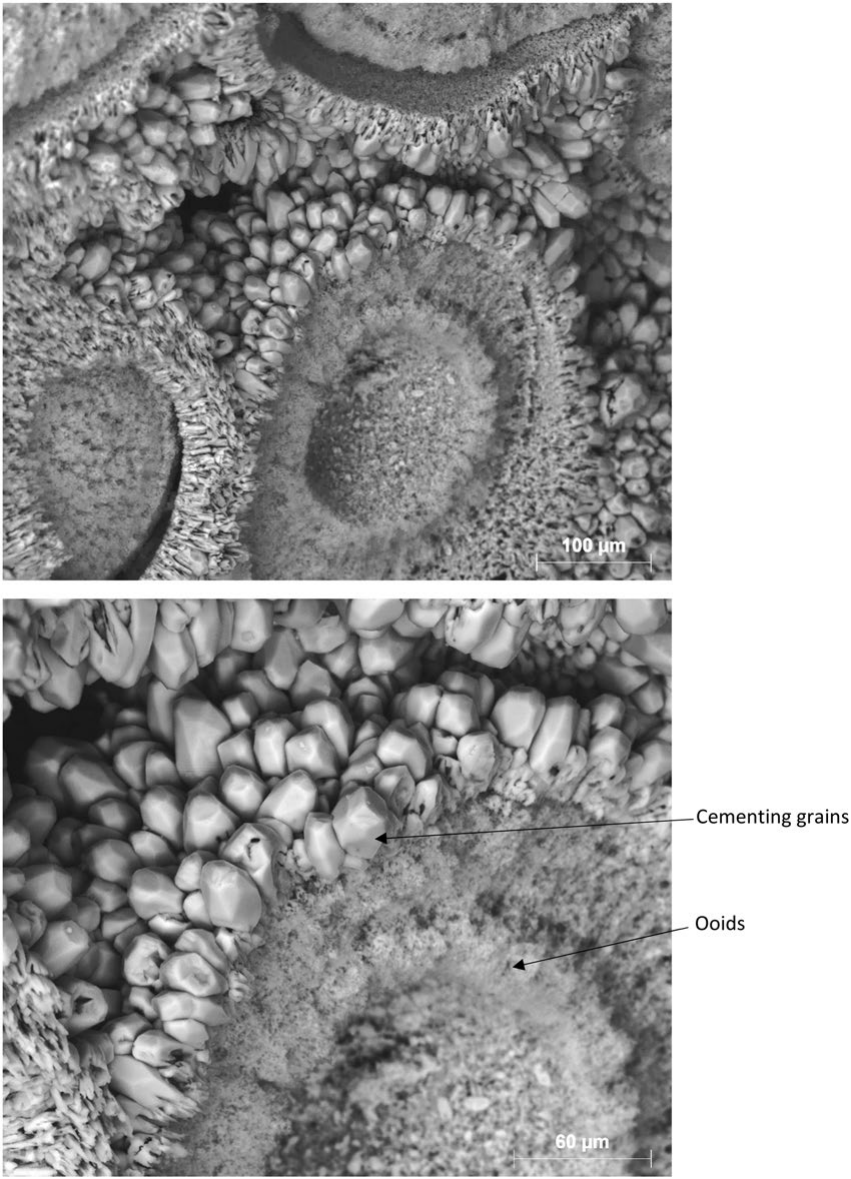
After ooids become accessible to carbonated brine, their dissolution occurs faster than the cementing grains.

**Figure 13:**
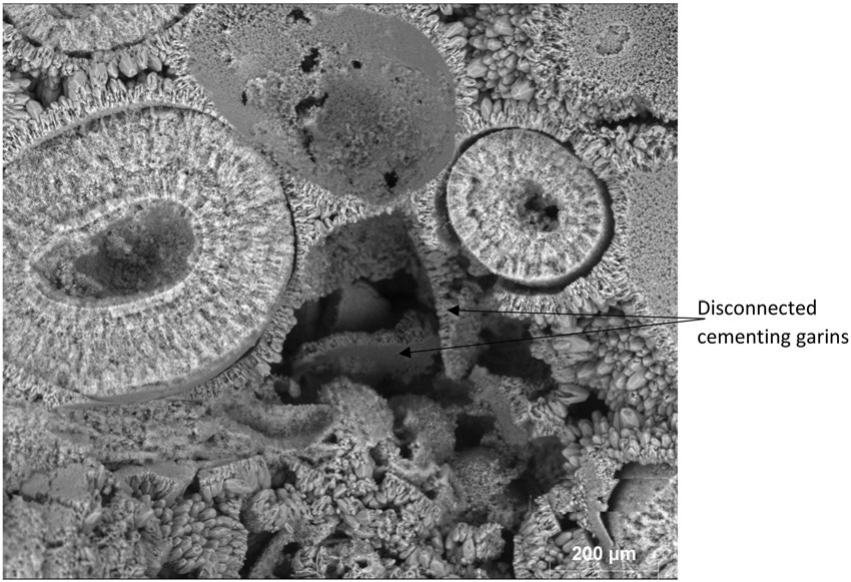
Faster dissolution of ooids led to the disconnection of the cementing grains and shell and their precipitation.

Based on SEM observations, during carbonated brine injection step, the pore body of Core A evolved initially through the dissolution of cementing calcite crystals, which in turn led to an increase in primary intergranular porosity. The calcite crystals originally had a rounded or angular geometry as shown in Fig. 10. Carbonated brine injection led to a heterogeneous dissolution of calcite crystals which caused a large increase in surface roughness of crystals as observed in Fig. 11A,B. The heterogeneous dissolution caused the formation of irregular micro-voids on the crystals which gradually coalesced and formed larger voids and a new type of intra-granular micro-porosity (Fig. 11). It should be noted that, due to sub-micron sizes of these voids, micro-CT resolution of a few microns would not typically capture their features. At later stages of the dissolution, the grain is reduced to a highly porous aggregate. Finally, the cementing calcite crystal completely dissolves and the ooids became accessible to the acidic brine and consequently, the dissolution of their cortical layers occurred (Fig. 12). Interestingly, as soon as the ooids became accessible to the carbonated brine, due to the higher surface contact area of the ooid cortex as opposed to the cements, they dissolved faster. In three-dimensional space, this phenomenon at some point leads to the disconnection of the cementing grains and shells and their transportation by the flow or their deposition in a pore body (Fig. 13). Ultimately, these calcite grains dissolve in the carbonated brine or block some pore throats.

**Figure 14:**
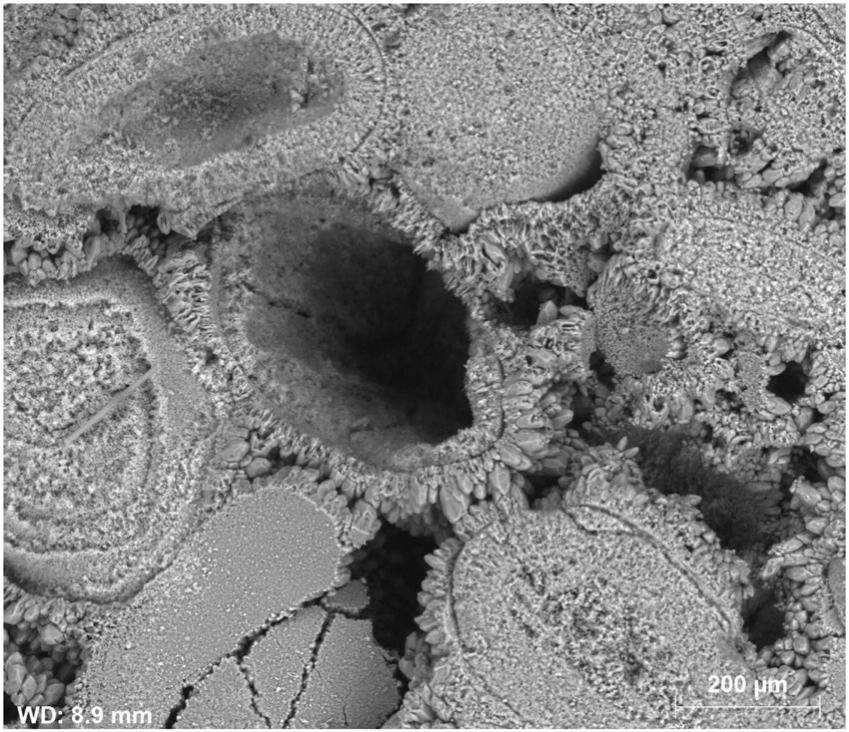
Evolution of moldic porosity due to availability of ooids to carbonated brine after the dissolution of cementing calcite crystals.

**Figure 15:**
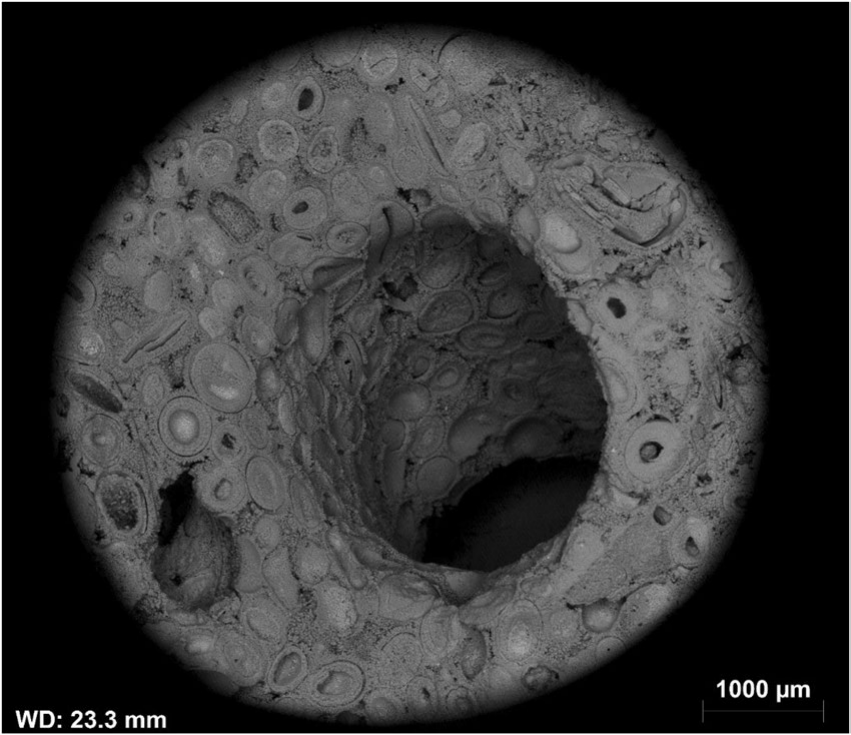
A big wormhole formed due to dissolution of the grains and ooids at the presence of carbonated brine.

**Figure 16:**
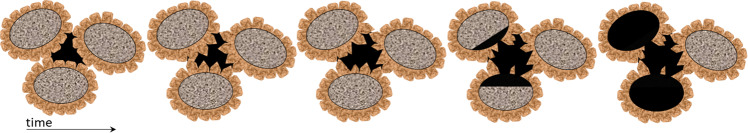
Schematic of the evolution in calcite cemented ooid grainstone porosity during the acidic brine injection- pore volume is shown in black color.

As a result of ooid dissolution, a series of isolated empty spaces (oomolds) and therefore a moldic porosity was evolved during carbonated brine injection (Fig. 14). It was found that for two isolated oomolds next to each other, if the dissolution of cementing calcite crystals continues, the isolated oomolds can merge and form a bigger pore and eventually a wormhole as shown in Fig. 15. The schematic of this evolution in calcite cemented ooid grainstone porosity during the acidic brine injection is shown in Fig. 16. These findings add new insights into the formerly works^13,34^ studied carbonated brine-carbonate rock reactions.

These pore structure changes not only influence the porosity type and porosity value of rocks, but they also affect the key flow properties of rocks including absolute and relative permeabilities^11,35^. The effects of fluid-rock interactions are therefore consequential for injectivity and residual trapping of CO_2_. Furthermore, such strong dissolution of calcite cementing grains and ooids, and subsequent separation of cementing calcite crystals lead to rock weakening and compaction, which at some point can negatively influence rock permeability and injectivity or in severe cases can lead to wellbore collapse. As such, having a thorough understanding of these pore structure alterations during CO_2_ injection into underground formations is essential.

#### Core B

**Figure 17:**
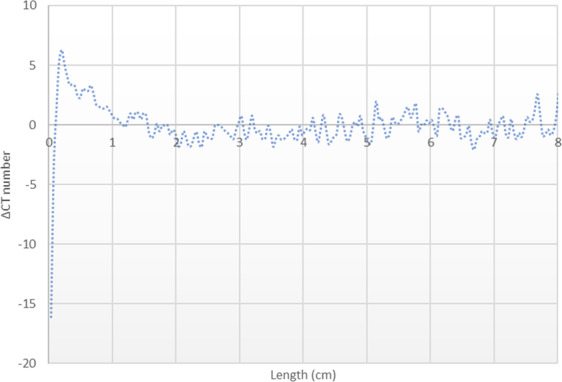
X-ray CT number gradient across the length of Core B shows minor mineral precipitation at very near core inlet face and no significant change in the rest of core.

As for Core A, X-ray CT images of Core B were obtained before and after the carbonated brine injection step and the results were compared to obtain the CT number gradient across this core (Fig. 17). Core B density and thus the CT attenuation number, except at very near inlet face, remained unaffected by the total flux of around 10 pore volumes of the carbonated brine injection period. The results indicate that the sudden local pressure drop at near Core B inlet caused minor calcite precipitation at the very near inlet side of the Core B followed by no significant change in the rest of the core. Minor precipitation very near to the inlet face did not influence the core’s fluid conductivity (i.e., absolute permeability).

**Figure 18:**
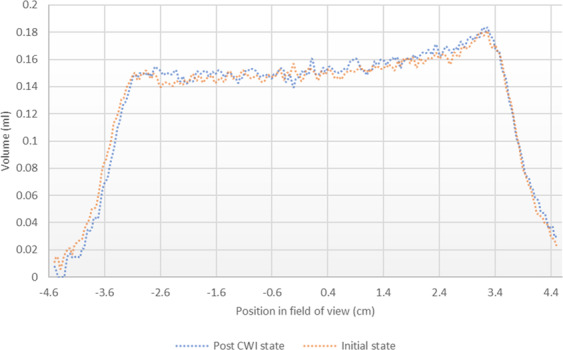
NMR T_2_ saturation profiles across the length of Core B pre and post carbonated brine injection step show no detectable change within the margin of error.

**Figure 19:**
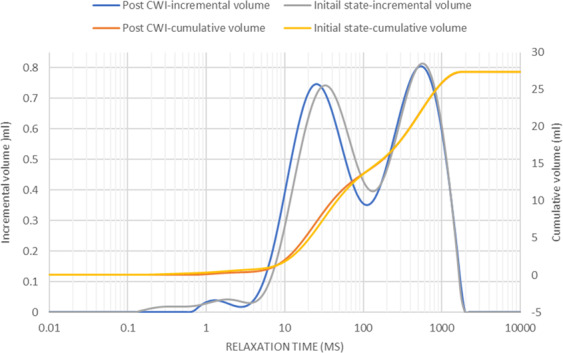
NMR T_2_ relaxation time profiles of Core B taken pre and post carbonated brine injection step indicate no change in medium and large size pore populations.

The minor precipitation near to the core inlet face did not result in any significant effect on the saturation profile or pore volume of the core measured before and after carbonated brine injection (Fig. 18). The NMR T_2_ profiles (Fig. 19) of this core taken pre and post carbonated brine injection indicate a negligible change in the population of large and medium-size pores while we had 47% reduction in micro-pores (T_2_ < 3 ms) population. Furthermore, while there is no change in the relaxation time of large pores, the relaxation time of medium size pores slightly shifted toward lower times. This can be either due to small increase in surface roughness of the pores caused by calcite dissolution or small decrease in pores volumes due to precipitation. Since these changes were small, they had no impact on T_2_ relaxation time of large pores as opposed to medium size pores. The reduction in population of micro-pores can be due to precipitation, however, since compared to medium and large size pores, micro-pores only made a very small portion of the core pore volume, they have minimum impact on rock permeability.

**Figure 20:**
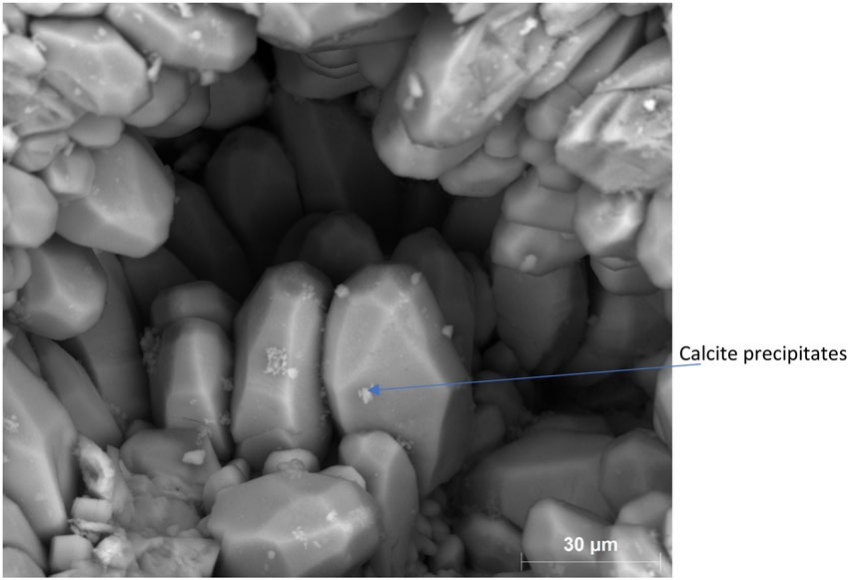
Calcite precipitates at the Core B inlet face initiated from the local pressure drop of carbonated brine front saturated with calcium.

The SEM data from the core inlet face indicates the presence of very minor precipitates of <1 micron sized calcite crystals (Fig. 20). Due to the sub-micron size of these precipitates, they could block the micro-pores which explain the observed reduction in their population from NMR T_2_ profile. Therefore, based on the obtained medical X-ray CT, NMR, and SEM data, the local pressure drop of around 250 psi on carbonated brine enriched with calcium had minor impact on Ca^2+^ and HCO_3_^−^ ions solubility in the carbonated brine and caused minor calcite precipitation. Therefore, a pressure reduction of this extent in the reservoir would be expected to have minimum to no impact on calcite precipitation.

### Scaling problem

Inductively coupled plasma-optical emission spectrometry analysis of the effluents during the experiment was conducted. The results indicate an increase in the Ca^2+^ concentration of the effluent brine during the carbonated brine injection step which is due to calcite dissolution. Furthermore, during the carbonated brine injection step, strong precipitation occurred in the outflow tube where the pressure dropped to atmospheric pressure. SEM data of the precipitates confirm that they are calcite deposits. As opposed to the 250 psi pressure drop in the composite core, the strong pressure drop from test pressure to atmospheric pressure had a very strong impact on disturbing the chemical equilibrium of the brine and caused scaling issues. This shows that the extent of calcite precipitation is a function of the amount of pressure drop. Calcite scale can precipitate on the surfaces of production piping and valves, damaging the equipment.

### Discussion

Based on the results from Core A, strong calcite dissolution is expected to occur in the near-wellbore region, which leads to the formation of wormholes and consequent increase in the porosity especially permeability of the near-wellbore region. Dissolution also leads to an increase in pore body roughness of the rock in the near-wellbore region and consequent formation of intra-grain micro and moldic porosity, at least for the case of calcite cemented ooid grainstones. The strong dissolution increases the pore throat sizes and even completely merges big pores together (Fig. 15) which reduces the capillary trapping of the non-wetting phase (CO_2_) and therefore decreases the residual trapping potential of the CO_2_ in this region. This means that more CO_2_ can freely migrate toward the top of the reservoir which consequently could increase the risk of CO_2_ leakage through caprock compared to a situation where flow is more diffuse. The decrease in capillary snap off of the non-wetting phase also leads to better connectivity of the non-wetting phase and therefore increases its relative permeability in the near-wellbore region. On the other hand, at a specific water saturation, when the pore body roughness increases, more water will be present in cracks, crevices, and corners of the pores which are connected by a thin film of water. Therefore, for a water-wet reservoir like saline aquifers, increase in roughness at near-wellbore regions could lead to a reduction in wetting phase conductivity and water relative permeability. It should be noted that these alterations can only be extended to near-wellbore regions where dissolution occurs strongly and the amount of precipitation is negligible.

Based on the results of Core B, which represents the situation far from wellbore regions, since carbonated brine already reaches equilibrium with calcite at near-wellbore region (Core A), its capacity for dissolving minerals decreases and therefore a minimum change in pore structure and pore body roughness due to calcite dissolution will occur in the interior of the reservoir. Furthermore, a reduction in the pressure of the carbonated brine front from injection pressure to reservoir pressure that occurs at distances far from wellbore region disturbs the equilibrium and causes the formation of calcite precipitation at some frontal position. The amount of this precipitation is a function of the amount of pressure drop and degree of enrichment of the carbonated brine front with calcium and bicarbonate ions. In the case of our experiments, the amount of precipitation induced by around 250 psi pressure drop was negligible and did not cause any change in rock pore structure, porosity and permeability. Pressure drop can also occur due to other reasons such as leakage of CO_2_ to lower pressure zones, or brine withdrawal for reservoir pressure balance or stopping the CO_2_ injection. The geochemical consequences of these pressure gradients should also be factored into predictions of the reservoir performance.

It should be noted that during CO_2_ injection into saline aquifers or hydrocarbon reservoirs, we may have two-phase or three-phase flow in porous media and this will affect the extent of reactions and their impacts on pore structure. Furthermore, the presence of an oil layer on the pore surface (i.e., oil-wet conditions) is expected to influence the reaction impacts on pore structure. These areas have not been considered in this work and require further investigation.

## Conclusion

A new procedure for reactive transport fluid flow tests was introduced in this study to assess the changes in hydraulic and pore structure of host carbonate formations during CO_2_ injection when host rock interacts with the CO_2_-saturated brine. From the initial test on a sample of Savonnieres limestone we observed the following:

1. Very strong calcite dissolution occurs close to the point of injection (near wellbore region) which leads to the formation of wormholes, increasing in porosity and permeability of the region and increasing the fluid injectivity.
2. Heterogenous dissolution of the grains in this upstream region increases intergranular porosity, increases pore body roughness, forms intra-grain micro-pores and micro-porosity, forms moldic porosity in grainstones, increases pore throats and pores connectivity and alters pore populations.
3. Increase in pore throats and pores connectivity due to strong calcite dissolution decreases the capillary snap off of the non-wetting phase and therefore we would expect to see decreases in residual trapping potential of the CO_2_ in the near-wellbore region in granular carbonate formations.
4. Since carbonated brine can equilibrate with calcite in the near-wellbore region, at distances far from the injection well, fluid-rock interactions become weak and therefore a minimal change in rock hydraulic properties and pore structure and populations are to be expected.
5. Reduction in the pressure of carbonated brine owing to flow gradients or rate changes can disturb the system equilibrium and lead to calcite precipitation. However, the amount of this precipitation in our proof-of-concept experiment where the pressure was dropped by 250 psi was negligible and thus its effects on pore structure, porosity, and permeability of the system were practically undetectable.
6. Our new procedure can be adapted to replicate the expected flow conditions and gradients in the near-wellbore or far into the reservoir, and should be helpful to predict the consequences of different fluid-rock interactions that can be encountered during various CO_2_ injection operations in the field.
